# Role of Stress Response Genes in Resistance and Germination of *Bacillus subtilis* Spores

**DOI:** 10.3390/microorganisms14040805

**Published:** 2026-04-01

**Authors:** Paula Gómara, Emma Pinilla, Jorge Bellón, Elisa Gayán

**Affiliations:** Department of Animal Production and Food Science, Faculty of Veterinary, AgriFood Institute of Aragon (IA2), University of Zaragoza—CITA, Miguel Servet 177, 50013 Zaragoza, Spain; pgomara@unizar.es (P.G.); emmapinilla00@gmail.com (E.P.); jbellon@unizar.es (J.B.)

**Keywords:** bacterial spores, *Bacillus subtilis*, cellular stress response, SigB activity, heat shock proteins, spore heat resistance, spore germination

## Abstract

Bacterial adaptation to hostile environments depends on the coordinated expression of stress response genes. When adverse conditions persist and nutrients become limiting, sporulating species may initiate sporulation as a last-resort survival strategy. However, sporulation under such conditions may alter the resistance and germination properties of the resulting spores. In this study, we investigated whether stress response regulators that facilitate vegetative cell adaptation to temperature and/or salinity changes during growth can influence the properties of *Bacillus subtilis* 168 spores. To this end, we examined the resistance and germination of mutant spores lacking key regulators of stress response pathways (SigB, SigW, SigX, Fur, HrcA, CtsR, and CssRS regulon), all produced under optimal sporulation conditions. The constitutive activation of the SigB-mediated general stress response, achieved through the deletion of its negative regulator RsbX, reduced spore heat resistance by 2.2-fold compared to the parental strain, while no effect was observed in vegetative cells. Additionally, Δ*rsbX* spores displayed both impaired nutrient-induced and CaDPA-induced germination. Collectively, these findings suggest that stress response regulators can influence spore behavior, although their effects may differ from those observed in vegetative cells.

## 1. Introduction

Exposure to environmental stresses triggers coordinated changes in gene expression that support bacterial survival and growth under adverse conditions. In sporulating bacteria, when stress continues and nutrients become limiting, sporulation serves as a last-resort strategy to ensure persistence. Spores are metabolically dormant, highly resistant to multiple stresses relative to vegetative cells, and can germinate into actively growing cells under favorable conditions [1,2]. Hostile sporulation environments can alter spore resistance and germination, with the direction and magnitude of these effects depending on the type and intensity of the stress [3]. In previous studies with *B. subtilis* 168, increasing the sporulation temperature above the optimum enhanced nutrient-induced germination, whereas lowering the sporulation temperature impaired germination and sensitized spores to heat. Increasing the salinity of the sporulation medium compromised nutrient-induced germination while enhancing spore heat resistance [4,5,6].

Understanding the mechanisms that modulate heterogeneity in spore behavior induced by varying sporulation conditions is critical, as such variability complicates the design of effective spore-control strategies. This is particularly relevant in food processing, where surviving and germinating spores may cause spoilage and raise food safety concerns when pathogenic *Bacillus* and *Clostridium* spp. are involved [7,8]. Behavioral variations induced by changes in sporulation conditions have been associated with alterations in key spore components—including core water, mineral, and dipicolinic acid (DPA) levels—and structural proteins [9,10,11,12,13]. However, the precise regulatory pathways underlying these effects remain unclear. In this context, adaptive responses in the mother cell required to maintain homeostasis and support spore development under adverse conditions may contribute to shaping the final spore phenotype [14,15].

Stress-response-related genes are often controlled by alternative RNA polymerase sigma factors [16]. In *B. subtilis*, the sigma B (SigB)-dependent general stress response is the best-known stress regulatory system. Under optimal growth conditions, SigB is activated by nutrient limitation upon entry into the stationary phase, driving the expression of hundreds of genes involved in stress adaptation, virulence, and cell differentiation, including sporulation, and thereby influencing spore morphogenesis and properties [14,17,18]. At both suboptimal and supraoptimal temperatures [19,20], as well as under subinhibitory salt concentrations [21], SigB has been shown to remain continuously upregulated in growing cells, and it is required during later developmental stages for efficient sporulation under adverse low-temperature conditions [22].

Additional sigma factors involved in responses to temperature and salinity shifts in *B. subtilis* include extracytoplasmic function (ECF) sigma factors, which play a central role in maintaining cell envelope homeostasis [23]. In particular, the SigW regulon has been demonstrated to be induced during vegetative growth and spore outgrowth at high salinity [24,25,26], but it is downregulated at suboptimal temperatures [27]. By contrast, the SigX-dependent response was repressed during growth at high salinity [21,24], whereas its presence enhanced the heat resistance of exponentially growing cells [28].

Besides alternative sigma-dependent genes, other stress-related responses can independently contribute to cellular adaptation, such as the expression of heat shock proteins (HSPs) against proteotoxic stresses [29]. The growth of *B. subtilis* at high temperatures or high salinity conditions has been shown to upregulate multiple HSP-encoding genes, including those from the HrcA, CtsR, and CssRS regulon [20,25,30,31]. Specifically, with respect to salt stress, the ferric uptake regulator (Fur) regulon is induced to maintain intracellular iron pools, compensating for the reduced iron availability caused by high-salinity conditions [21,25,32].

Although stress responses are well characterized in the context of cellular adaptation to harsh growth conditions, their effects on resulting spore properties remain poorly understood. This study aims to assess the role of stress response genes—particularly those triggered by temperature and salt concentration shifts—in spore resistance and germination, using well-characterized deletion mutants with altered sigma factor-dependent pathways (SigB, SigW, or SigX), HSP expression, or Fur-dependent regulation.

## 2. Materials and Methods

### 2.1. Strains and Sporulation Conditions

The *B. subtilis* strains used in this study are listed in Table 1. Based on previous studies, knockout mutants were chosen, targeting genes reported to upregulate or downregulate specific regulons in *B. subtilis* vegetative cells, as evidenced by gene/protein expression analyses and/or phenotypic characterization (Table 1). Knockout mutants were originated from the *B. subtilis* genome-scale deletion library BKE [33] and were supplied by the National BIO-Resource Project (Japan), while the wild type (WT) strain was provided by the Bacillus Genetic Stock Centre (Columbus, OH, USA). Strains were stored at −80 °C in NB (Nutrient Broth No. 2; Oxoid, Madrid, Spain) supplemented with 25% of glycerol (Panreac, Barcelona, Spain). When indicated, the antibiotic resistance cassette was excised through the transient introduction of the temperature-sensitive plasmid pDR244, constitutively expressing Cre recombinase [33], to rule out polar effects as the cause of phenotypic differences between mutant and WT spores. Each gene disruption was confirmed via PCR, with primer pairs attaching outside the target region, and further verified by sequencing (Macrogen, Madrid, Spain).

For revitalization, strains were grown on NAYE plates (Nutrient Agar supplemented with 0.6% Yeast Extract; Oxoid, Madrid, Spain). In the case of mutant strains, the medium was supplemented with 1 μg/mL erythromycin and 12.5 μg/mL lincomycin [33]. For the cultivation of vegetative cells, one single colony was inoculated in a 60 mL flask containing 10 mL of NB, and it was incubated overnight at 37 °C with shaking (130 rpm; Promax 1010, Heidolph Instruments GmbH & Co. KG, Schwabach, Germany). For sporulation, 20 μL of the vegetative cell culture was inoculated into 200 mL flasks containing 20 mL of Schaeffer’s medium (2xSG) with some modifications, as described by Freire et al. [5]. Sporulation flasks were incubated at 37 °C for 36 h, when the maximum spore yield was reached.

Spores were harvested via centrifugation (4000× *g*, 20 min, 4 °C; Megafuge 1.0R, Heraeus, Hanau, Germany), and the pellet was washed three times with distilled water. Spores were then purified via buoyant density centrifugation using Nycodenz^®^ (Serumwerk, Bemburg, Germany), as previously described [5,53]. After purification, the spores were washed twice with distilled water (6000× *g*, 2 min, 25 °C) and then stored at −20 °C until use. Phase-contrast microscopy (Nikon Eclipse E400, Nikon Inc., Tokyo, Japan) was used to verify that the suspensions contained at least 98% phase-bright spores. To assess biological variability, three independent spore batches were prepared for each strain.

### 2.2. Heat Inactivation

Heat treatments were carried out in a thermoresistometer TR-SC [54]. Briefly, this device consists of a 450 mL chamber equipped with an electrical heating element, a cooling coil, an agitation device to guarantee sample and temperature homogenization, and ports and valves for sample injection and extraction. This system is pressurized with nitrogen gas (2.0 bars) to perform treatments over 100 °C and facilitate sample extraction.

For spore treatment, suspensions were adjusted to an optical density at 600 nm (OD_600_) of ca. 1.0 (ca. 1 × 10^8^ spores/mL). For vegetative cell treatment, cells collected upon entry into the stationary phase and prior to the initiation of sporulation were adjusted to ca. 1 × 10^8^ cells/mL. A volume of 0.2 mL of each sample was injected into the treatment chamber once the treatment medium (McIlvaine citrate-phosphate buffer at pH 7.0 [55]) had been pre-warmed to the desired temperature (97.5 °C, 100.0 °C, 102.5 °C, or 105.0 °C for spores and 56.0 °C for vegetative cells; ±0.1 °C). Aliquots of 0.2 mL were collected at different time points and pour-plated on NAYE. Plates were incubated at 37 °C for 24 h, after confirming that longer incubation times did not affect survival counts. Colony counts were obtained using an automatic colony-counting system based on image analysis. The limit of quantification was 20 CFU/mL.

### 2.3. Modeling of Heat Inactivation Curves

Inactivation curves were obtained by plotting the survival fraction (Log_10_ (*N*_t_/*N*_0_), where *N*_0_ and *N_t_* represent the number of survivors (CFU/mL) at time 0 and after different treatment intervals, respectively) as a function of treatment time. Curves were fitted to the Log-linear + shoulder equation developed by Geereaerd et al. [56] (Equation (1)) using GraphPad PRISM 8.4.2 (GraphPad Software Inc., San Diego, CA, USA). In this model, the kinetic parameters are *Sl* (min), which represents the shoulder duration before the onset of exponential inactivation, and K_max_ (min^−1^), which represents the slope of the exponential decay. Additionally, the coefficient of determination (R^2^) and the root mean square error (RMSE) were calculated to assess the goodness of fit.

(1) $${\text{Log}}_{10}\;N_{t}={\text{Log}}_{10}\;N_{0}-\;\frac{K_{max}\cdot t}{\text{Ln}\;10}+{\text{Log}}_{10}\left(\frac{exp^{K_{max}\cdot Sl}}{1+\left(exp^{K_{max}\cdot Sl}-1\right)e^{-K_{max}\cdot t}}\right)$$

To compare the resistance among strains, the 3D_T_ parameter was estimated as an indicator of the time needed for 3 Log reductions at each treatment temperature. To evaluate the relationship between heat resistance and temperature, thermal death time (TDT) curves were obtained by plotting Log_10_ (3D_T_) values against temperature. z values were calculated as the inverse of the slope, representing the temperature increase required to reduce the 3D_T_ value by one Log_10_ cycle.

### 2.4. Germination Assay

Spores at a final OD_600_ of ca. 0.5 (ca. 5 × 10^7^ spores/mL) were incubated with 10 mM L-alanine (Sigma-Aldrich, St. Louis, MO, USA), 10 mM L-valine (Sigma-Aldrich), an AGFK mixture (L-asparagine (Amresco Inc., Solon, OH, USA), D-glucose (Panreac), D-fructose (Panreac), and KCl (Panreac); 10 mM each component) or 50 mM DPA chelated with calcium (CaDPA; 1:1 DPA [Sigma-Aldrich] and CaCl_2_ [VWR International Chemicals, Delaware County, PA, USA]) prepared in a 25 mM HEPES buffer (pH 7.4; Sigma-Aldrich). Germination was evaluated by monitoring the decrease in OD_600_ resulting from DPA release, and spore hydration over 4 h at 37 °C. OD_600_ was measured using a multiwell plate reader (CLARIOstar^®^ Plus, BMG LABTECH, Ortenberg, Germany), with readings automatically recorded every 3 min. Between measurements, plates were shaken orbitally for 30 s to prevent spore sedimentation.

Germination efficiency at the end of the 4 h assays was determined by counting germinated (phase-dark/gray) and dormant (phase-bright) spores (100–150 cells per sample) using phase-contrast microscopy (Nikon Eclipse E400, Nikon Inc., Tokyo, Japan) (Equation (2)). The lower and upper limits of quantification for germinated spores were approximately 6.0% and 97.0%, respectively.

(2) $$\text{Germination Efficicency}\;\left(\%\right)=\frac{\text{No. phase-dark/gray cells}}{\text{No. phase-dark/gray}\;+\;\text{No. phase-bright spores}}\times \;100$$

### 2.5. Modeling of Germination Curves

Germination curves were constructed by plotting the percentage decrease in OD_600_ (OD_t_/OD_i_ × 100, where OD_i_ and OD_t_ represent the initial value and the value measured at further incubation times, respectively) over time. Germination curves were fitted to the Plateau Followed by One-Phase Decay model (Equation (3)) using GraphPad PRISM 8.4.2. According to this model, the *lag* parameter (min) represents the time before the onset of exponential OD_600_ decay, the *plateau* corresponds to the endpoint value of the percentage decrease in OD_600_, and *k* (min^−1^) represents the germination rate constant. The coefficient of determination (R^2^) and the root mean square error (RMSE) were estimated to assess the goodness of fit.

(3) OD_600_ fall (%) = (100 − *plateau*)*e*^(−^
*^k^*^(^*^t^*
^−^
*^lag^*^))^ + *plateau*

### 2.6. Statistical Analysis

Statistical analyses were performed with the unpaired parametric *t*-test using GraphPad PRISM 8.4.2. Differences with *p* ≤ 0.05 were considered statistically significant. The figures show the means and standard deviations from at least three biological replicates.

## 3. Results

### 3.1. Effect of Stress Response Genes on Spore Resistance to Heat

To evaluate the role of the selected stress response genes in heat resistance, inactivation curves of the corresponding knockout mutants and the WT spores were obtained at different temperatures (97.5 °C, 100.0 °C, 102.5 °C, and 105.0 °C). Most survival curves exhibited an initial shoulder, likely reflecting a balance between damage accumulation and repair mechanisms prior to the log-linear decay phase [5,57,58]. Consequently, heat resistance parameters were defined as the shoulder length (*Sl*) and the inactivation rate (*K_max_*), calculated by fitting the inactivation data to the Log-linear + shoulder model (Equation (1)) (Table 2 and Table S1). To compare heat resistance and its thermal dependence, 3D_T_ values were calculated and used to construct TDT curves.

**Figure 1:**
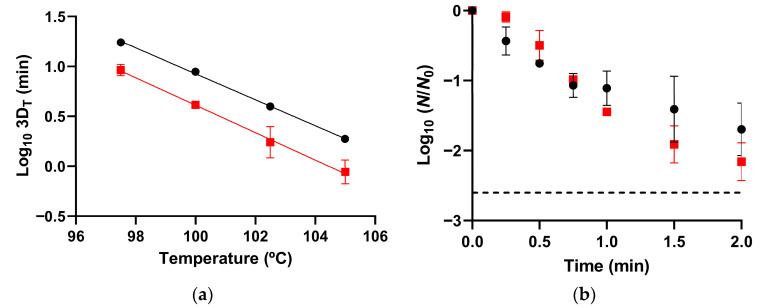
(**a**) Thermal death time (TDT) curves of WT (●) and Δ*rsbX* (■) spores obtained over a temperature range of 97.5 to 105.0 °C. (**b**) Survival curves of WT (●) and Δ*rsbX* (■) vegetative cells at 56.0 °C. Values represent the means of two biological replicates, and error bars indicate the standard deviation. The dotted line indicates the limit of quantification.

Among the mutants tested, the ∆*rsbX* mutant—lacking a negative regulator of SigB activity—showed the greatest change in spore heat resistance compared to the WT strain. The absence of the *rsbX* gene resulted in a 2.2-fold reduction in 3D_T_ values between 97.5 °C and 105.0 °C (*p* ≤ 0.05; Figure 1a, Table 2). Consistently, ∆*rsbX* spores showed z values similar to those of WT spores (7.30 ± 0.53 °C and 7.70 ± 0.17 °C, respectively; *p* > 0.05). The higher sensitivity of the ∆*rsbX* spores was mainly associated with a reduced shoulder length, together with an increased inactivation rate at the lowest temperatures tested (97.5 °C and 100.0 °C; *p* ≤ 0.05; Table 2). These differences in heat inactivation kinetics between ∆*rsbX* and WT spores were also observed after the removal of the antibiotic resistance marker from the mutant strain (*p* ≤ 0.05). In contrast, ∆*sigB* spores exhibited similar heat resistance to WT spores (*p* > 0.05; Table S1).

Replacement of the *rsiW* gene with an erythromycin resistance cassette, encoding the anti-SigW factor, but not *sigW* itself, as well as disruption of *sigX*, reduced spore heat resistance at certain temperatures (*p* ≤ 0.05; Table S1). However, these differences vanished after the removal of the antibiotic resistance cassette (*p* > 0.05). Regarding HSP regulators and the Fur regulator, all Δ*hrcA*, Δ*ctsR*, Δ*cssR*, and Δ*fur* spores showed 3D_T_ values similar to those of the WT spores at all temperatures tested (*p* > 0.05; Table S1).

We also assessed the effect of deleting each gene in the heat resistance of vegetative cells prior to the initiation of sporulation and compared it with that observed in the corresponding spores. Notably, despite presumably increased SigB activation, Δ*rsbX* cells were equally sensitive to WT cells at 56 °C (Figure 1b).

### 3.2. Effect of Stress Response Genes on Spore Germination

**Figure 2:**
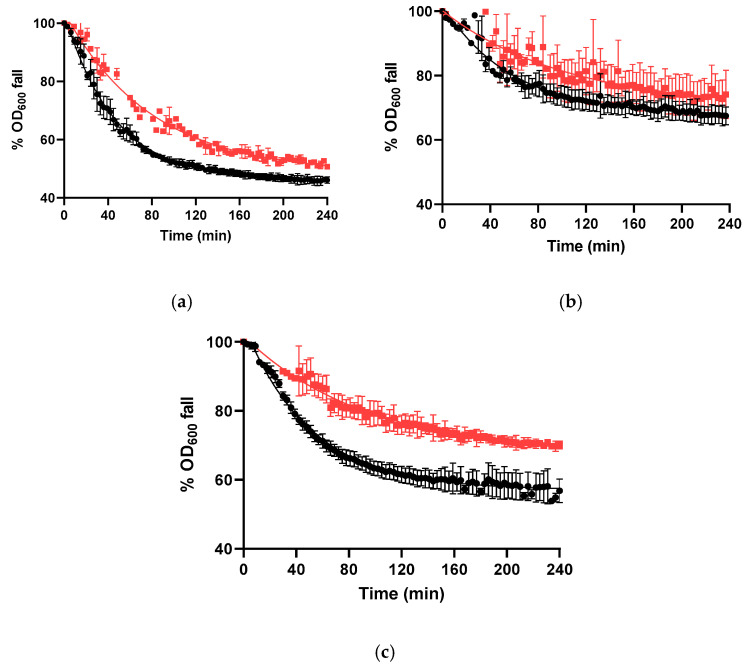
Germination curves of WT (●) and Δ*rsbX* (■) spores in the presence of L-valine (**a**), AGFK (**b**), or CaDPA (**c**). Values represent the means of three biological replicates, and error bars represent the standard deviation.

Germination of mutant and WT spores was induced by nutrients acting through distinct germinant receptors (GRs): L-valine, acting via GerA, and AGFK, acting via GerB and GerK [2]. For comparison, germination kinetics parameters (lag phase, *lag*, and germination rate, *k*) were determined from the decrease in OD_600_ over 4 h, and endpoint germination efficiencies were assessed using phase-contrast microscopy. In the WT strain, L-valine triggered more efficient germination than AGFK (Figure 2, Table 3), consistent with previous reports [6]. The Δ*rsbX* mutant was the only strain that maintained significantly altered germination compared with the WT strain after the removal of the antibiotic resistance marker. More specifically, this mutant displayed reduced germination rates in L-valine and AGFK (Figure 2, Table 3; *p* ≤ 0.05). However, Δ*rsbX* spores only showed reduced germination efficiency in L-valine.

We also tested germination with the chemical CaDPA, which triggers germination independently of GRs by activating the cortex-lytic enzyme ClwJ [59]. While the germination efficiency of WT spores was above the limit of quantification, the proportion of germinated Δ*rbsX* spores remained significantly lower (*p* ≤ 0.05; Figure 2c, Table 3).

It should be noted that the replacement of the *hrcA*, *sigW*, or *fur* gene with an erythromycin resistance cassette resulted in changes in germination rate and/or efficiency in response to the tested nutrient germinants; however, these differences disappeared after the cassette was removed.

## 4. Discussion

We investigated whether stress responses mediated by SigB, SigW, SigX, HrcA, CtsR, CssR, and Fur—known to support growth adaptation under adverse conditions—also influence spore properties in *B. subtilis* 168 by analyzing spores from mutants lacking the principal regulatory genes of each system, produced under optimal sporulation conditions. Most notably, the modulation of SigB activity during sporulation had a substantial impact on spore behavior. Specifically, upregulation of SigB activity, through deletion of its negative regulator RsbX [36,37,38], reduced spore heat resistance at all temperatures tested. In addition, derepressed SigB activity impaired spore germination both through GR activation by nutrients and via GR-independent ClwJ activation by CaDPA. However, the extrapolation of these findings to other *Bacillus* spp. should be approached with caution. Although the core components of the SigB pathway (SigB, RsbV, and RsbW) are highly conserved among *Bacillus* spp., the regulatory network is not uniform across all members of the *Bacillales* [18]. For instance, the feedback regulator RsbX is absent in the *Bacillus cereus* group. Furthermore, predictions of SigB target genes indicate that they are not identical across species within the *Bacillales* or even among different *B. subtilis* strains [18]. Indeed, our findings contrast with previous observations in *B. subtilis* PS832 and *B. cereus* ATCC 14579, in which a *sigB* deletion mutant produced spores with increased heat sensitivity and, in the latter case, compromised nutrient-induced germination [14,15]. Therefore, this inter- and intra-specific plasticity of the SigB regulon may partly explain the phenotypic differences observed.

With respect to resistance, it is well established that, in vegetative cells, the loss of SigB increases sensitivity to multiple stresses [34,35], while the induction of SigB activity—either through transient exposure to sublethal stresses [34,60,61] or constitutive activation by mutations in *rsb* gene products from the SigB activation cascade, such as hyperactive *rsbU* alleles—enhances resistance [62]. However, in this study, ∆*rsbX* stationary-phase cells, prior to the initiation of sporulation, exhibited heat resistance comparable to that of the WT strain. Therefore, SigB derepression appeared not to affect vegetative cell heat resistance but reduced that of their progeny spores, whereas the loss of SigB increased vegetative cell sensitivity without affecting spore resistance in *B. subtilis* 168.

The detrimental effects of upregulated SigB activity in spores are consistent with the observation that the activation of this sigma factor activity, whether by stress exposure or synthetic induction, reduces sporulation efficiency, possibly as a mechanism to prevent compromised spore quality [22,63]. In fact, the sporulation efficiency of the ∆*rsbX* mutant was 1.8-fold lower than that of the WT strain. Mechanistically, the activation of SigB was demonstrated to induce the transcription of the *spo0E* gene, which encodes a phosphatase that inactivates the master sporulation regulator Spo0A-P, thereby contributing to reduced sporulation commitment [63,64]. Nevertheless, it should be noted that additional SigB-independent mechanisms may also contribute to defective sporulation phenotypes under specific stresses [64,65]. For instance, Widderich et al. [65] reported that high salinity hampers the association of SigH with the core RNA polymerase, which consequently blocks the completion of sporulation.

Understanding the reasons why the Δ*rsbX* strain produces spores with impaired heat resistance and nutrient-induced germination compared to the WT strain requires further physicochemical characterization of the spores. Increased core water content, together with reduced levels of core minerals and DPA, has been associated with decreased heat resistance [12,66,67]. In addition, the composition of the spore coat, cortex, and inner membrane has been shown to modulate heat resistance and germination [68,69,70,71]. The SigB regulon includes genes involved in lipid and membrane protein remodeling, as well as peptydoglycan restructuring [18], which may affect the properties of the spore’s inner membrane and cortex.

Regarding other stress-related regulators, the lack of effect from disrupting their encoding genes on spore properties appears largely uncoupled from the effects observed in vegetative cells, similar to what was observed for the SigB network. For instance, disruption of the *sigX* gene did not affect heat resistance, even though previous studies have shown that loss of SigX activity increases sensitivity in vegetative cells [28]. In the case of HSPs, upregulation of the HrcA or CtsR regulon, as well as downregulation of the CssRS regulon, did not affect spore resistance. Consistent with these observations, loss-of-function mutations in the *hrcA* or *ctsR* gene did not affect the recovery of heat-treated *B. subtilis* PS832 spores [15]. In addition, the increased heat resistance of spores resulting from transient exposure to a temperature upshift during sporulation could not be attributed to the upregulation of HSPs [15,72]. Although an *hrcA* deletion mutant has shown improved adaptation to temperature upshifts during vegetative growth [49], in this study, sustained upregulation of HrcA-dependent chaperones until sporulation initiation did not affect the heat resistance of vegetative cells, as observed in spores.

This study represents an attempt to identify stress response regulators that may impact spore properties. However, the effects observed here do not straightforwardly mirror the behavior of spores produced under adverse environmental conditions. Indeed, the changes in resistance and germination exhibited by the examined mutants compared to the WT strain do not fully match those reported under stressful sporulation conditions. For instance, in previous studies using a comparable experimental setup, sporulation at suboptimal or supraoptimal temperatures—conditions that sustain SigB activity during vegetative growth and sporulation [19,20,22] and that would be expected to negatively affect spore resistance and germination, as observed in the Δ*rsbX* mutant—resulted in spores with reduced heat resistance and impaired germination under suboptimal conditions, but heat resistance remained unchanged and germination was enhanced under supraoptimal conditions [5,6]. Several factors may explain these discrepancies.

First, because stress response activation is influenced by several factors—such as strain and stress intensity [19,20,73,74]—it is essential to confirm, for each specific sporulation condition, that the stress responses of interest are fully activated in the mother cell and can therefore be transmitted to the resulting spores. Second, the mutants experienced abnormally extensive and constitutive regulatory changes that do not reflect the strength and timing of naturally induced environmental stress responses. For instance, the SigB regulon is typically activated transiently due to the activity of negative regulators such as RsbX [75], although it has been reported that increased SigB levels can be maintained during prolonged incubation under subinhibitory adverse temperatures or salinity [19,20,21]. Additionally, the strength of the SigB-dependent response depends on the intensity of the stress signal [17,76] Furthermore, the aberrant stress response regulation caused by the complete absence of a specific regulator may create imbalances among interacting components within the same system or across other networks, thereby altering overall cell fitness, as observed with the deletion of SigB-negative modulators [75]. Third, under adverse temperature or salinity conditions, the activation of multiple stress-related regulons—each exerting different effects on spores—can coexist and interact. Fourth, the pleiotropic effects of such stresses—beyond those caused by activation of stress responses—may mask or compensate for changes in the expression of stress-related genes. For example, alterations in spore coat structure, which have been associated with the increased heat resistance of spores produced under high salinity [9], may help buffer the potential side effects of upregulated stress responses. Finally, as a consequence of cell-to-cell heterogeneity in the expression of stress-related genes and in the decision to initiate sporulation [77,78,79,80], the fraction of cells that succeed in sporulating may have previously experienced lower levels of stress response regulators than the average population—for example, reduced SigB activity [63,64]—thereby circumventing their impact on the resulting spores.

## 5. Conclusions

This study demonstrates that the SigB-dependent general stress response can influence the resistance and germination properties of *B. subtilis* spores, although these effects do not mirror those observed in vegetative cells. Constitutive activation of SigB—via deletion of its negative regulator RsbX—reduced spore heat resistance and impaired both nutrient-induced and CaDPA-induced germination. In contrast, regulatory changes in the SigW, SigX, HrcA, CtsR, CssR, or Fur regulons, which contribute to cellular growth adaptation under environmental stress, did not affect spore behavior.

Although engineered upregulation of SigB contributes to shaping spore properties, it does not necessarily explain the spore traits observed under adverse sporulation conditions. This discrepancy likely arises from differences in the extent and timing between environmental and synthetic modulation of SigB activity, the resulting network imbalances, the coexistence and interaction of multiple stress regulons alongside unrelated pleiotropic effects, and cell-to-cell heterogeneity in the expression of stress-related and sporulation genes. Further studies will be required to elucidate how stress response networks interact with spore structural and biochemical determinants under environmentally relevant conditions.

## Figures and Tables

**Table 1:** Bacterial strains used in this study.

Strain	Genotype	Description	Evidence of the Effect of Gene Deletion on Vegetative Cells
168	*trpC2*	Wild-type strain.	
Δ*sigB*	*trpC2* Δ*sigB::erm*	168 lacking the RNA polymerase sigma factor B (SigB), involved in the general stress response.	The lack of the *sigB* gene increases sensitivity to multiple stresses, including heat and oxidative agents [34,35]
Δ*rsbX*	*trpC2* Δ*rsbX::erm*	168 lacking the protein serine phosphatase RsbX, which participates in the negative feedback loop that maintains or restores low SigB activity following induction.	The lack of the *rsbX* gene upregulates SigB activity [36,37,38].
Δ*sigW*	*trpC2* Δ*sigW::erm*	168 lacking the RNA polymerase ECF-type sigma factor W (SigW), involved in the response to cell-wall-active antibiotics and alkaline stress.	The lack of the *sigW* gene increases sensitivity to cell-envelope-targeting antimicrobials [39,40,41].
Δ*rsiW*	*trpC2* Δ*rsiW::erm*	168 lacking the anti-SigW, which sequesters SigW to prevent its function.	The lack of the *rsiW* gene upregulates SigW activity [42,43].
Δ*sigX*	*trpC2* Δ*sigX::erm*	168 lacking the RNA polymerase ECF-type sigma factor X (SigX), involved in the response to cationic antimicrobial peptides.	The lack of the *sigX* gene increases sensitivity to heat, hydrogen peroxide, and cell-envelope-targeting antimicrobials [28,39,41,44].
Δ*fur*	*trpC2* Δ*fur::erm*	168 lacking the transcriptional repressor Fur, involved in iron homeostasis.	The lack of the *fur* gene upregulates siderophore biosynthesis and iron uptake [45,46,47].
Δ*hrcA*	*trpC2* Δ*hrcA::erm*	168 lacking the transcriptional repressor HrcA of heat-shock genes.	The lack of the *hrcA* gene upregulates GroESL and DnaK-GrpE-DnaJ chaperones and favors growth adaptation following a temperature upshift [48,49].
Δ*ctsR*	*trpC2* Δ*ctsR::erm*	168 lacking the transcriptional repressor CtsR of heat-shock genes.	The lack of the *ctsR* gene upregulates Clp family proteases [50,51].
Δ*cssR*	*trpC2* Δ*cssR::erm*	168 lacking the CssR factor from the two-component transcriptional activator CssRS of heat-shock genes.	The disruption of CssRS complex upregulates extracytoplasmatic HtrA-family chaperone-type proteases [31,52].

**Table 2:** Heat resistance parameters (*Sl*, *Kmax*, and 3D_T_) obtained after fitting inactivation curves to the Log-linear + shoulder model (Equation (1)) for WT and Δ*rsbX* spores at different treatment temperatures. Data in brackets represent the standard deviations of the mean values calculated from three biological replicates. An asterisk indicates statistically significant differences (*p* ≤ 0.05) between the two strains for each parameter at a given treatment temperature.

Strain	Temperature (°C)	*Sl* (min)	*K*_max_ (min^−1^)	3D_T_ (min)	R^2^	RMSE
WT	97.5	6.36 (0.49)	0.63 (0.05)	17.80 (0.49)	0.987	0.107
	100.0	2.73 (0.58)	1.18 (0.14)	8.95 (0.42)	0.987	0.113
	102.5	0.76 (0.03)	2.30 (0.07)	3.93 (0.30)	0.989	0.135
	105.0	0.27 (0.05)	4.61 (0.38)	1.98 (0.10)	0.982	0.116
Δ*rsbX*	97.5	0.59 (0.60) *	0.81 (0.10) *	9.26 (1.18) *	0.970	0.165
	100.0	0.15 (0.27) *	1.74 (0.06) *	4.13 (0.30) *	0.985	0.147
	102.5	0.06 (0.05) *	4.37 (1.79)	1.81 (0.60) *	0.978	0.152
	105.0	0.05 (0.08) *	8.79 (2.73)	0.90 (0.26) *	0.989	0.125

**Table 3:** Germination kinetics parameters (*lag* and *k*) obtained after fitting germination curves to the Plateau Followed by One-Phase Decay model (Equation (3)), and the percentage of germinated spores determined microscopically at the end of the assay (4 h; Equation (2)) for WT and Δ*rsbX* spores in different germinants. Values in brackets correspond to standard deviations of the means calculated from three biological replicates. An asterisk indicates statistically significant differences (*p* ≤ 0.05) between the two strains for each parameter under a given germinant.

Germinant	Strain	*lag* (min)	*k* (min^−1^)	R^2^	RMSE	Germination Efficiency (%)
L-valine	WT	11.32 (7.08)	0.0380 (0.0127)	0.972	1.90	93.90 (2.23)
	Δ*rsbX*	18.25 (8.59)	0.0136 (0.0044) *	0.980	2.00	88.14 (5.76) *
AGFK	WT	14.98 (9.57)	0.0222 (0.0014)	0.955	1.60	37.20 (3.73)
	Δ*rsbX*	9.26 (4.46)	0.0123 (0.0051) *	0.842	2.54	33.11 (8.91)
CaDPA	WT	9.35 (4.48)	0.0233 (0.0056)	0.977	1.15	≥97.00 ^1^
	Δ*rsbX*	30.52 (11.59)	0.0192 (0.0036)	0.984	1.00	91.56 (0.85)

^1^ One or more values were higher than the maximum limit of quantification (≥ 97.00%).

## Data Availability

The original contributions presented in this study are included in the article/Supplementary Material. Further inquiries can be directed to the corresponding author.

## Supplementary Materials

The following supporting information can be downloaded at https://www.mdpi.com/article/10.3390/microorganisms14040805/s1. Table S1: Heat resistance parameters (*Sl*, *Kmax*, and 3D_T_) obtained after fitting inactivation curves to the Log-linear + shoulder model (Equation (1)) for the indicated strains at different treatment temperatures. Data in brackets represent the standard deviations of the mean values calculated from three biological replicates. An asterisk indicates statistically significant differences (*p* ≤ 0.05) between WT and each mutant for a given parameter at each treatment temperature.
