# Supplementary material for: Role of Stress Response Genes in Resistance and Germination of Bacillus subtilis Spores
Source: Microorganisms. 2026 Apr 1;14(4):805. doi: 10.3390/microorganisms14040805 (PMC13118429; doi:10.3390/microorganisms14040805)
Supplement: Supplementary file 1 [file microorganisms-14-00805-s001.zip › microorganisms-4182543-supplementary.pdf]

Table S1. Heat resistance parameters (*Sl*, *Kmax*, and $3D_T$) obtained after fitting inactivation curves to the Log-linear + shoulder model (Equation 1) for the indicated strains at different treatment temperatures. Data in brackets represent the standard deviations of the mean values calculated from three biological replicates. An asterisk indicates statistically significant differences ($P \leq 0.05$) between WT and each mutant for a given parameter at each treatment temperature.

Strain	Temperature (°C)	*Sl* (min)	K_{max} (min^{-1})	$3D_T$ (min)	R^2	RMSE
WT	97.5	6.36 (0.49)	0.63 (0.05)	17.80 (0.49)	0.987	0.107
	100.0	2.73 (0.58)	1.18 (0.14)	8.95 (0.42)	0.987	0.113
	102.5	0.76 (0.03)	2.30 (0.07)	3.93 (0.30)	0.989	0.135
	105.0	0.27 (0.05)	4.61 (0.38)	1.98 (0.10)	0.982	0.116
Δ*sigB*	97.5			ND		
	100.0	2.10 (0.85)	1.08 (0.23)	8.69 (1.22)	0.985	0.128
	102.5			ND		
	105.0	0.30 (0.16)	4.28 (0.49)	1.93 (0.28)	0.991	0.104
Δ*sigW*	97.5	7.14 (0.73)	0.40 (0.07)**	24.99 (4.09)*	0.978	0.114
	100.0	2.03 (0.78)	0.85 (0.20)	10.38 (2.43)	0.995	0.067
	102.5			ND		
	105.0	0.29 (0.11)	3.99 (1.19)	2.13 (0.64)	0.990	0.089
Δ*rsiW*	97.5	3.96 (1.30)*	0.68 (0.12)	14.39 (0.55)*	0.987	0.101
	100.0	1.73 (0.67)	1.21 (0.10)	7.45 (0.22)*	0.993	0.077
	102.5	0.23 (0.24)*	2.56 (0.23)	2.95 (0.37)*	0.993	0.057
	105.0	0.22 (0.13)	6.08 (0.44)*	1.36 (0.08)*	0.992	0.085
Δ*sigX*	97.5	4.13 (2.02)	0.52 (0.02)*	17.52 (1.94)	0.988	0.089
	100.0	0.46 (0.31)*	0.79 (0.04)*	9.22 (0.76)	0.990	0.068
	102.5	0.20 (0.35)	2.14 (0.78)	3.72 (0.93)	0.980	0.100
	105.0	0.00 (0.01)*	5.24 (0.58)	1.33 (0.14)*	0.988	0.077
Δ*fur*	97.5	7.10 (1.29)	0.58 (0.12)	19.40 (1.88)	0.998	0.042
	100.0	3.28 (0.38)	0.97 (0.13)	10.46 (1.17)	0.994	0.057
	102.5	1.15 (0.18)*	1.73 (0.12)*	5.17 (0.10)*	0.982	0.078
	105.0	0.23 (0.24)	2.93 (0.49)*	2.63 (0.40)	0.986	0.074
Δ*hrcA*	97.5	6.17 (1.56)	0.44 (0.03)*	21.92 (1.77)*	0.994	0.067

	100.0	2.59 (0.58)	1.04 (0.33)	9.68 (1.80)	0.986	0.149
	102.5			ND		
	105.0	0.35 (0.10)	4.00 (1.55)	2.25 (0.75)	0.988	0.118
Δ*ctsR*	97.5	4.64 (1.26)	0.49 (0.02)*	18.85 (1.70)	0.993	0.082
	100.0	1.31 (0.58)*	0.81 (0.21)	10.29 (2.42)	0.990	0.130
	102.5			ND		
	105.0	0.15 (0.23)	3.91 (1.14)	2.03 (0.48)	0.974	0.168
Δ*cssR*	97.5			ND		
	100.0	1.02 (0.80)*	0.88 (0.16)	9.94 (0.93)	0.988	0.134
	102.5			ND		
	105.0	0.25 (0.09)	5.98 (1.32)	1.44 (0.27)*	0.990	0.113

ND. Not determined, as no significant differences were observed at the other two tested temperatures.
